# Statistics of a Sharp GP2Y Low-Cost Aerosol PM Sensor Output Signals

**DOI:** 10.3390/s20236707

**Published:** 2020-11-24

**Authors:** Klemen Bučar, Jeanne Malet, Luca Stabile, Jure Pražnikar, Stefan Seeger, Matjaž Žitnik

**Affiliations:** 1 Jožef Stefan Institute, Jamova 39, 1000 Ljubljana, Slovenia; matjaz.zitnik@ijs.si; 2 Jožef Stefan International Postgraduate School, Jamova 39, 1000 Ljubljana, Slovenia; 3 Faculty of Mathematics and Physics, University of Ljubljana, Jadranska 19, 1000 Ljubljana, Slovenia; 4 Institut de Radioprotection et de Sûreté Nucléaire (IRSN), PSN-RES, SCA, 91190 Gif-Sur-Yvette, France; jeanne.malet@irsn.fr; 5 Department of Civil and Mechanical Engineering, University of Cassino and Southern Lazio, 03043 Cassino, Italy; l.stabile@unicas.it; 6 Institute Andrej Marušič, University of Primorska, Muzejski Trg 2, 6000 Koper, Slovenia; jure.praznikar@upr.si; 7 Faculty of Mathematics, Natural Sciences and Information Technologies, University of Primorska, Glagoljaška 8, 6000 Koper, Slovenia; 8 BAM—Federal Institute for Materials Research and Testing, 12489 Berlin, Germany; stefan.seeger@bam.de

**Keywords:** low-cost sensors, aerosol sensors, air quality sensors

## Abstract

In this work, we characterise the performance of a Sharp optical aerosol sensor model GP2Y1010AU0F. The sensor was exposed to different environments: to a clean room, to a controlled atmosphere with known aerosol size distribution and to the ambient atmosphere on a busy city street. During the exposure, the output waveforms of the sensor pulses were digitised, saved and a following offline analysis enabled us to study the behaviour of the sensor pulse-by-pulse. A linear response of the sensor on number concentration of the monosized dispersed PSL particles was shown together with an almost linear dependence on particle diameters in the 0.4 to 4 micrometer range. The gathered data about the sensor were used to predict its response to an ambient atmosphere, which was observed simultaneously with a calibrated optical particle counter.

## 1. Introduction

Simple particulate matter (PM) sensors are gaining popularity due to their low price, easy handling and good temporal resolution, despite difficulties with obtaining accurate quantitative results. There are several such sensors on the market. One of them, Sharp GP2Y1010AU0F, is a popular choice for a low-cost PM monitoring due to its high availability and simple interfacing. It is used in several low-cost PM appliances, such as TSI AirAssure [1] and UB AirSense [2] and, due to its low power, small mass and good temporal resolution, it is suitable for a drone assisted [3] and an indoor spatio-temporal monitoring of PM distributions [4,5]. Numerous educational and do-it-yourself projects can be found on the World Wide Web employing popular small microcontrollers [6]. It has been a subject of several scientific studies and calibration projects [7,8,9,10,11,12,13,14] (and references therein), where good correspondence with real PM concentration was demonstrated. The sensor is potentially suitable for low-power applications as it operates in a pulsed mode. However, none of the aforementioned works focused on the raw readout of the sensor. The manufacturer’s documentation [15] only explains the basic interfacing to the sensor and lacks the description of the internal working principles and the method of transformation of raw data into airborne particle mass concentration. Carefully observing the output of the sensor, one can find some inconsistencies in manufacturer’s instructions, which will be addressed later in the paper.

## 2. Sensor Description

**Figure 1:**
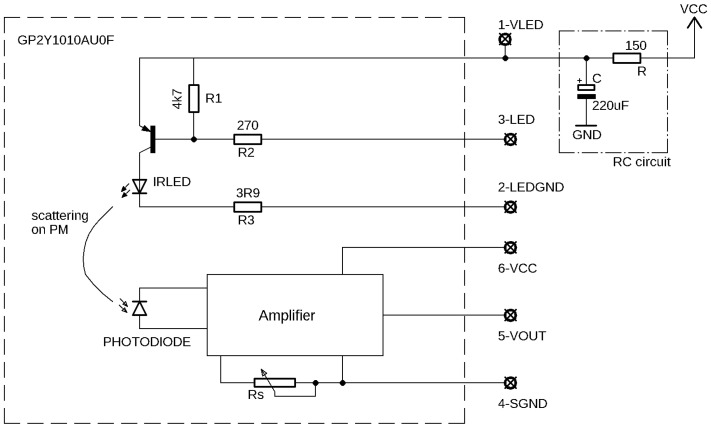
Sensor schematics with manufacturer’s recommended RC circuit. Redrawn with permission from the Data sheet [15]. The values of the elements R1—R3 are measured on one specific sensor and are not given by the manufacturer.

The GP2Y sensor [15,16] consists of an infrared light emitting diode transmitter (IRLED), a photodiode receiver and an amplifier circuit (Figure 1). The amplifier circuit includes a band pass filter. It filters out light intensity changes on a scale slower than milliseconds to avoid sensitivity to the variation of the ambient light. The circuits contain several elements without listed specifications which would be crucial to know to understand the inner working of the sensor. For example, manual measurement of the value of the IRLED current limiting resistor (R3 in Figure 1) gives 3.9 Ω. The small resistance allows a very large current through the IRLED (≈1 A), which can be damaging to the IRLED unless it is temporarily limited to a very short current pulse.

**Figure 2:**
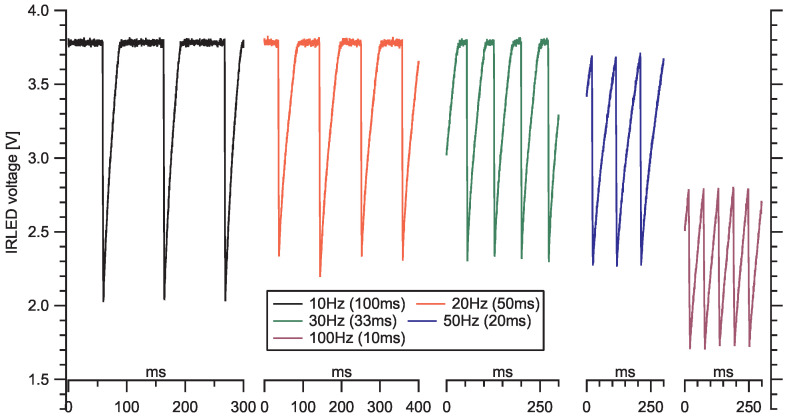
Observed V-LED driving voltage at different driving frequencies: (from left to right) 10, 20, 30, 50 and 100 Hz. Rightmost (100 Hz) is the manufacturer’s recommended driving frequency.

The manufacturer therefore requires an external RC protection circuit to be applied by the user, where the direct current is additionally limited with the resistor R after the capacitor C is discharged (Figure 1). However, the application of the protection circuit limits the working frequency of the sensor in the pulsed mode operation. At the beginning of each pulse (when input 3-LED goes low), fully charged capacitor C is discharging a high current—limited with R3 = 3.9 Ω resistor—through the IRLED which provides a short and intense light pulse, after which the capacitor is (partly) discharged. It starts recharging after the pulse is completed and, to reach full charge, it needs a few tens of milliseconds since τ = RC = 33 ms (for the manufacturer, recommended values of *R* = 150 Ω and *C* = 220 μF). However, the next pulse is recommended by the manufacturer already after 10 ms, while the capacitor is not recharged yet. Therefore, the IRLED power input (pin 1-VLED) can remain below full voltage (Figure 2), depending on the pulsing frequency and RC constant. Moreover, this voltage fluctuates if any of the three parameters (*f*, *R*, *C*) fluctuate (e.g., with temperature), which can manifest in a fluctuation of the emitted and the scattered light intensity and is reflected in the stability of the detected signal. With lower driving frequency (less than 30 Hz), the voltage on the input capacitor manages to recover between pulses and we expect the amplitude of each pulse to be higher and more stable. The manufacturer claims that the two elements (resistor and capacitor) are mandatory for the operation of the sensor, and the sensor does not work without them. We would, however, characterise them rather as a *protection circuit*. In any case, we find the protection RC circuit recommended by the manufacturer inconsistent with sampling instructions.

Another unexplained feature is a trimmer potentiometer that is accessible on the sensor through the hole in the casing (denoted Rs in Figure 1) and is marked as *sensitivity adjustment*. According to the manufacturer, it is set during the calibration in the factory and should not be changed by the user. There are no further details given about the function of this element. During our test, we have kept its position unchanged as was set in the factory.

**Figure 3:**
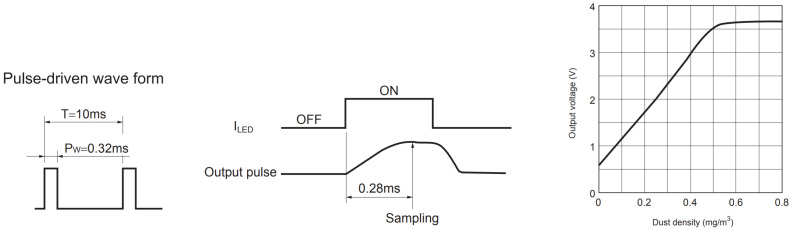
Manufacturer’s recommended driving signals timings and calibration curve taken with permission from the Data sheet [15]. (**Left**) the control TTL signal on pin 3-LED, (**center**) predicted output pulse shape and moment of the recommended voltage sampling. The sampled voltage should be correlated to the dust density as shown in the given calibration curve (**right**).

The manufacturer’s instructions on the interpretation of the sensor readout are found in the Data sheet [15]. The voltage of the analog output pulse, coincident with the user-driven IRLED pulse, should be sampled 280 μs after the start of the user pulse. The voltage at that particular moment can be converted to an aerosol mass concentration in μg/m${}^{3}$ in a chart given in the Data sheet (Figure 3, right). It is not obvious which property of the pulse is of measuring interest; however, based on the given data (Figure 3, center), it might be the peak voltage of the output pulse. In this case, another approach is feasible in which the detailed shape of the output pulse is sampled, and the peak voltage is reconstructed mathematically, allowing pulse time jitter corrections and pulse shape observation.

In most of the published studies on this sensor, the manufacturer’s instructions were strictly followed. Li and Biswas [12] used the manufacturer’s readout recipe. According to their description, 10-s intervals were averaged, i.e., 1000 pulses, though it is not clear what the sampling interval refers to: “*In these experiments, the sampling interval of the microcontroller (Arduino) was set to 2.5 s, and every four samples were averaged before being sent to the computer through XBee. Therefore, the log file stored on the computer recorded signal every ten seconds.*” It is also not clear whether the recommended resistor and capacitor were used at all (see Figure 1 in [12]). Wang et al. [9] give no specific implementation information. We assume they followed manufacturer’s instructions; however, the 30 s window is mentioned. Li et al. [5] state: “*Sampled voltage 28 ms after the leading edge*” (probably a typing error, should be 0.28 ms), “*driven by a square wave of 32 ms pulse width*” (probably a typing error, should be 0.32 ms); however, they used a low frequency of 4 Hz sampling: “*Sampling interval is 0.25 s.*” The averaging window was 1 s, which corresponds to four pulses. Later, the 29th order finite-duration impulse response low pass digital filter was used to smooth the data but preserves 1 s resolution. Hapidin et al. [17] used the manufacturer’s instructions: “*The IR-LED of GP2Y sensor should be driven by a square wave voltage having the width of (0.32 ± 0.02) ms and the period of (10 ± 1) ms.*” None of the studies mentioned any discrepancy of the sensor behaviour not corresponding to the manual, except for the calibration suggestion (Figure 3, right). It is clear that none of the studies relied on the manufacturer’s calibration, and it was explicitly marked as inconsistent in Sousan et al. [11].

## 3. Device Description

**Figure 4:**
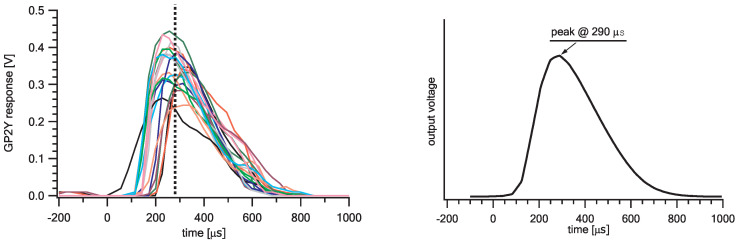
(**Left**) An example set of twenty consecutive recorded real output pulses. The black dotted line corresponds to t = 0.28 ms; (**Right**) the overall average shape of many (several thousands) pulses differs from the manufacturer’s prediction (see Figure 3, center).

To analyse the sensor’s output pulses in details, a dedicated device for saving the measured pulse waveforms was built at JSI. The Raspberry Pi (RPi) is used as a central processor for data acquisition, storage and communication. The IRLED input of the sensor (pin 3-LED in Figure 1, active low) is triggered with the digital output of the RPi. The sensor’s analog output (pin 5-VOUT in Figure 1) is digitized by Microchip’s ADC MCP3008 [18] connected via the SPI bus. The sensor’s output recording starts 300 μs before the trigger signal is issued and is continually sampled with approximately 100 kSps (one point each 10 μs) for another 1.2 ms. Each point of the pulse is timestamped with a microsecond accuracy relative to the trigger pulse and is stored in a compressed HDF5 file [19] on an RPi SD card. Data sampled before the trigger signal (negative times in Figure 4) are used to establish the zero baseline.

A total of about 150 voltage points are sampled during each pulse at 10-bit ADC resolution within the total unipolar ADC range of 0 to 5 V. The frequency of pulse triggering is configurable, though usually set to 10 Hz, causing a total dataflow of three kilobytes per second. The final HDF file size with all the metadata saved is about 400 megabytes per day. Each pulse is Unix timestamped. Due to the lack of a real time clock on RPi boards, a battery powered RTC module DS1302 is added to the device and is synchronised over Wi-Fi when available. The expected clock drift without network synchronisation is approximately 20 s/week.

Simple online analysis is performed in real time and the results are displayed on an attached LCD for diagnostic purposes. A Wi-Fi network connection for remote control, diagnostics and RTC synchronization is utilised where available but is not required for the operation. The device works autonomously, and there is no user interaction required, except for the shutdown button due to the RPi safe shutdown procedure.

The GP2Y sensor is mounted outside the control box and is shielded from direct sunlight but exposed to a natural airflow as much as possible without any additional fans. Two almost identical devices were built and will be referred to as JSI1 and JSI2, the only difference being that the RPi models used were 1B and 3, respectively. The same in-house developed software was used in both devices. The calibration tests were performed only with JSI2; however, both devices were used simultaneously in the environmental campaign.

## 4. Results

Recording all of the output pulses of the sensor enabled later offline analysis of their shape and other properties. In Figure 4, we present an example of twenty consecutive output pulses recorded during two seconds in a clean atmosphere, where we expect identical pulses. Huge pulse to pulse variations are observed, the source of which is not clear. The average shape shows a sigmoidal-like start of the peak with linear decay immediately after the peak maximum, inconsistent with manufacturer description. The peak of the average shape is reached at 0.290 ms after pulse triggering.

**Figure 5:**
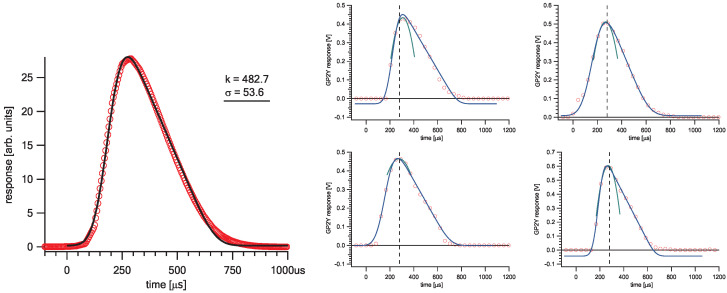
(**Left**) model function from Equation (3) with *k* = 482.7, σ = 53.6 (black solid) compared to a measured average pulse shape (red circles). (**Right**) some randomly chosen examples of individual real pulses successfully fitted with the model function (blue line, Equation (3)) and parabola through highest three points (green). The dashed vertical line corresponds to the manufacturer’s recommended sampling moment.

There are several different approaches to quantify a sampled pulse height. The manufacturer recommends sampling a single point at a fixed time of 280 μs after the pulse initiation. However, due to time jitter, this method does not always return the peak voltage of the pulse (assuming this is the relevant parameter) and is hence considered inappropriate for a precise pulse quantification. A better approach would be continuous sampling in an interval including the peak and calculating a second-order polynomial through the three points closest to the maximum. The pulse height is then quantified by the maximum of the obtained parabola. Such simple calculation can be applied in real time even by a low performance microcontroller (Figure 5, right). The three highest points describe the peak, but they do not contain any information about the shape of the pulse. To further analyse the pulse, one can construct an advanced function of the appropriate shape. We chose a convolution of a Gaussian function g(μ,σ) and a linear decay h(k), which models the average pulse shape quite well (see Equation (3) and Figure 5, left). It allows for a more detailed analysis of unevenly shaped pulses, as it is the case here. The linear function is limited with a Heaviside step function H(x) so that it decays from starting value 2/k at time zero to zero at time *k* with the slope $-2/k^{2}$, and is zero everywhere else:

(1) $$h\left(x;k\right)=\left(-\frac{2}{k^{2}}x+\frac{2}{k}\right)\;H\left(x\right)\;H\left(-\frac{x}{k^{2}}+\frac{1}{k}\right).$$

The area under h(k) is unity. The convoluted function with area *A* is

(2) $$y\left(t;A,k,\sigma \right)=A\;h\left(k\right)*g\left(0,\sigma \right)=\int_{-\infty }^{\infty }h\left(x;k\right)\;g\left(t-x;0,\sigma \right)\;dx,$$

(3) $$y\left(t;A,k,\sigma \right)=\frac{A}{k^{2}}\left(\left(k-t\right)\left(\mathrm{erf}\left(\frac{k-t}{\sqrt{2}\sigma }\right)+\mathrm{erf}\left(\frac{t}{\sqrt{2}\sigma }\right)\right)-\sqrt{\frac{2}{\pi }}\;\sigma \;e^{-\frac{{\left(k-t\right)}^{2}}{2\sigma^{2}}}\left(e^{\frac{k(k-2t)}{2\sigma^{2}}}-1\right)\right).$$

Fitting such a function to each output pulse provides additional control over the measuring process: badly shaped pulses returning unsuccessful fits or unacceptable goodness of fit (e.g., $\chi^{2}$) can be identified and discarded, as well as valid fits returning parameters out of limits. Average modern microprocessors are very capable of real-time fitting of a pulse model with the function like the one described above.

### 4.1. Sensor Response to a Clean Air and Low Pressure Chamber

In the absence of airborne particles in an active sensor’s volume, a constant repeatable output signal is expected. It is evoked by the light reflected and scattered from the plastic shield housing of the sensor. The variation of this signal is a measure of the inherent sensor noise, coming from the emission amplitude noise and detection noise. While we had no direct means to characterise the repeatability of the IRLED emitted light intensity, we were able to measure a current variation through IRLED to be less than 5%.

**Figure 6:**
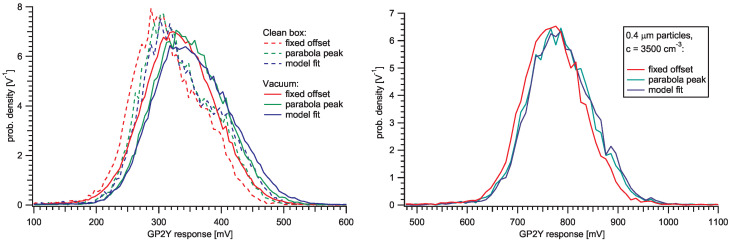
Pulse heights were determined with three different methods: with the recommended fixed-time offset, with fitting parabola over three highest points and with fitting the model function. The parameters of the distributions are gathered in Table 1. (**Left**) pulse height distributions in a clean box (*n* < 10 part./cm${}^{3}$) and in a low-pressure chamber (*p*≈ 20 mbar). Histograms include approx. 40,000 pulses each. (**Right**) pulse height distributions in a stable atmosphere with approx. 3500 particles per cubic centimeter dispersed in air. PSL particles of size 0.4 μm were used. Histograms include approx. 8000 pulses each. The duration of the measurement was about half an hour. See also the corresponding leftmost time graph in Figure 7.

To quantify the inherent sensor noise, we performed three measurements with JSI2 device. First, we put the sensor into a low-pressure chamber, which was evacuated to *p*≈ 20 mbar. Although we were unable to measure the PM distribution, we assume a relatively clean atmosphere. Second, we measured the pulse distribution in a controlled box at IRSN after several hours of filtering the air in the chamber and controlled the remaining PM distribution with a Welas instrument, which showed less than 10 particles per cubic centimeter. Estimating sensitive volume of the sensor to be a few cubic millimeters, we expect zero or one particle in the active volume for most of the time. Third, we analysed the pulse distribution during a stable and known atmosphere with high (≈150 μg/m${}^{3}$) concentration of 0.4 μm PSL spheres. The resulting distribution of output signal maxima is approximately described by a Gaussian distributions shown in Figure 6 with extracted parameters in Table 1. We used several different methods to extract the pulse peak parameter: manufacturer’s recommended fixed-time voltage sampling, calculation of parabola over three highest points and peak fit of the model pulse shape function. All three methods give similar results and confirm the unexpectedly huge pulse-to-pulse variation in amplitude and time. We attribute the major contribution of variation to the detection and amplification stage of the sensor, which is incorporated in an integrated circuit with unknown internal details. The results in Table 1 show that the width of the pulse distribution in the high particle concentration regime is similar to in the clean atmosphere (70–80 mV), which confirms the source of the inherent noise being in the detector and does not vary with the concentration of the PM in the air. The coefficient of variation (CV, defined by the ratio of standard deviation and mean) takes values in the range of 10–22% compared to 2–51% reported in [11].

It is interesting to note that the received signal was higher in vacuum than in the clean air, probably due to the scattering on the remaining particles in the low pressure chamber and higher light absorption in air.

The inherent noise exhibiting as huge pulse to pulse variations must be averaged out in order to obtain useful data. When calculating the average of *N* pulses from the same distribution, the absolute uncertainty of the average $\bar{X}$ is given by $\sigma /\sqrt{N}$. To keep the relative uncertainty of the average below *p*, at least

(4) $$N>{\left(\sigma /\left(\bar{X}p\right)\right)}^{2}$$

pulses must be averaged. For example, this corresponds to at least 100 pulses for *p* = 0.02, a time window of about 10 s at 10 Hz pulsing. The duration of the averaging window should be chosen based on the expected rate of change of the observed phenomena. On the other hand, if data about every pulse are saved, the averaging parameters can be conveniently chosen later at the offline analysis. Knowing the minimum number of pulses to achieve required accuracy must also be considered in low-powered (battery) applications, since the total number of pulses is limited with the available charge capacity.

From the determined widths of the pulse height distributions, one can calculate the limit of detection [9] $\mathrm{LOD}=3\sigma_{\mathrm{blk}}/k_{m}$, where $\sigma_{\mathrm{blk}}$ is the standard deviation at clean air (blank) conditions and $k_{m}$ is a sensitivity slope of the sensor’s linear response on different PM mass concentrations. It depends on the number of points averaged:

(5) $$\mathrm{LOD}=\frac{3\sigma }{k_{m}\sqrt{N}}.$$

Other quantifications of detection limits can be determined by using different coefficients instead of 3 (6 for limit of determination, 10 for limit of quantitation LOQ).

### 4.2. Sensor Response to Monodisperse Latex Test Aerosols with Known Particle Sizes and Number Concentrations

**Figure 7:**
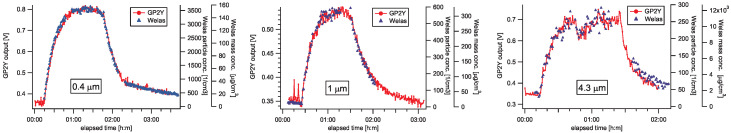
GP2Y sensor response (red, circles, left axis) to a known test aerosol compared to Welas particle number concentration measurement (blue, triangles, right axis) for different monosized atmospheres. Each datapoint represents the average of a 50 s time window (i.e., 500 pulses for GP2Y).

We measured the JSI2 sensor’s response to a well-defined monosized particle atmosphere. Commercial PSL latex spheres were dispersed into dried air and fed into the air chamber ($1.1\times 1.2\times 0.8\;m^{3}$) at the IRSN facility [20]. The particle number concentration and size distribution were continuously monitored every 50 s with a Welas particle spectrometer Digital 2100 [21]. PSL particles with manufacturer stated diameters of 0.39, 1.03, and 4.3 μm and density of 1.05 g/cm${}^{3}$ were dispersed using the aerosol nebulizers: Palas AGF 2.0 [22] for the two smaller sizes and the Collision Nebulizer [23] for the largest size. The Sharp sensor was triggered at 10 Hz and all pulses were digitized and saved for later analysis. A comparison of measured data for both instruments is shown in Figure 7, where the peak of each pulse is determined with a model function from Equation (3) and then averaged over 500 values to obtain 50 s time resolution, comparable with the Welas instrument. No pulse *shape* dependence after pulse amplitude normalization was observed when comparing pulses originating from different particle sizes. The amplitudes of recorded pulses were never above ≈1 V, regardless of the full sensor output range being ≈3.5 V (see Figure 1, right), for which we were able to achieve only by an introduction of a solid object in the sensor sensitive area to confirm the proper functioning of the DAQ circuit. In other works, most authors report raw GP2Y output in arbitrary units [5,9,12], so no comparison is possible. Hapidin et al. [17] (see Figure 10 and 11) report background voltage of about 1.25 V and measuring up to 3 V of output. Liu et al. [13] obtained 0.6–2.0 V with PM mass concentrations up to 1600 μg/m${}^{3}$. Sousan et al. [11] (see Figure 5B), on the other hand, obtained all outputs below 1 V. The reason for this discrepancy is not known to us, and one of the possible explanations is different factory calibration. We have not tested how the output of the sensor can be influenced by adjusting Rs sensitivity trimmer on the sensor itself.

Up to a scaling factor, the response of the sensor is comparable to the Welas readings. For each particle size *d*, linear correlations of the sensor’s fitted maxima *U* and the measured particle number concentration $c_{d}$ can be observed with the slopes $b_{d}$ depending on the size of the particles:

(6) $$U=U_{0}+b_{d}c_{d}.$$

**Figure 8:**
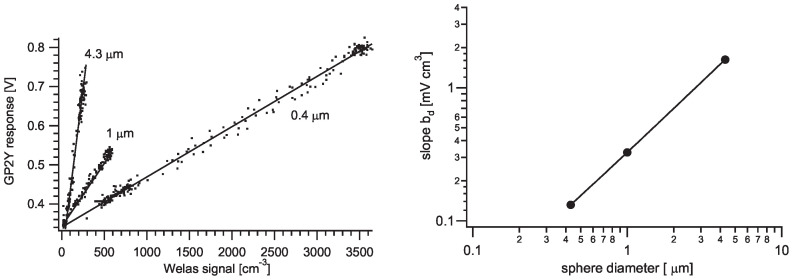
(**Left**) sensor output correlated with particle number concentration for different PSL particle sizes (the same data as in Figure 7). (**Right**) slope dependence on particle size shows best power fit $a\;{\left(d/d_{0}\right)}^{n}$ with power n=1.11±0.04 and a=(0.321±0.017) mV cm${}^{3}$, $d_{0}$ = 1 μm. Numerical results are in Table 2. The data from the Sharp sensor were averaged on the same time scale as the Welas instrument (i.e., 50 s, *N* = 500).

Considering the limitation of only three different particle sizes in this study, we can still try to estimate whether the response slope $b_{d}$ is linear with particles’ size (*d*), their cross section ($d^{2}$) or mass or volume ($d^{3}$). Fitting the $b_{d}=a{\left(d/d_{0}\right)}^{n}$ dependence, we obtain *n* = 1.10 (Figure 8 right and Table 2), suggesting that the slope $b_{d}$ is almost linearly dependent on particle diameter. The $d_{0}$ = 1 μm is introduced for unit consistency. Assuming addition of the signals from different particles, the sensor’s response *U* on polysized particles ensemble with known particle concentration distribution $c_{d}$ can be modelled as:

(7) $$U=U_{0}+a\sum_{d}c_{d}\;{\left(\frac{d}{d_{0}}\right)}^{n}.$$

The sum goes over all particle sizes *d* that are seen by the sensor and their diameters are within the validity of the used power-law, the limits of which we were unable to test. For known particle sizes, density and number concentrations, the mass concentrations can be calculated and Equation (7) can be modified. The slope is then $k_{m,d}=b_{d}/m_{0d}$, $m_{0d}$ being a mass of a single particle with the diameter *d*, mass concentration $\mu_{d}=c_{d}m_{0d}$ and $\tilde{a}=a/m_{0}$, $m_{0}=m_{0d}$ for $d=d_{0}$. The above equation suitable for work with mass concentrations is then:

(8) $$U=U_{0}+\tilde{a}\sum_{d}\mu_{d}\;{\left(\frac{d}{d_{0}}\right)}^{n-3}.$$

The limit of detection for different particle sizes can be estimated from Equation (5), where the *N* is given with the averaging time (*N* = 500 for 50 s window) and σ = 75 mV from Figure 6. The calculated LOD for our experimental conditions is given in Table 2. For comparison, Wang et al. report LOD = 26 μg/m${}^{3}$ for incense particles with mode size of 0.260 μm [9] (see supplementary material). Table 3 gives the summary of sensor’s parameters in studies where GP2Y was compared to different reference instruments. Slope and $R^{2}$ are quoted where possible for easier comparison with this work. A similar table for more parameters and more sensors can be found in [24].

### 4.3. Sensor Response to Ambient Aerosols

**Figure 9:**
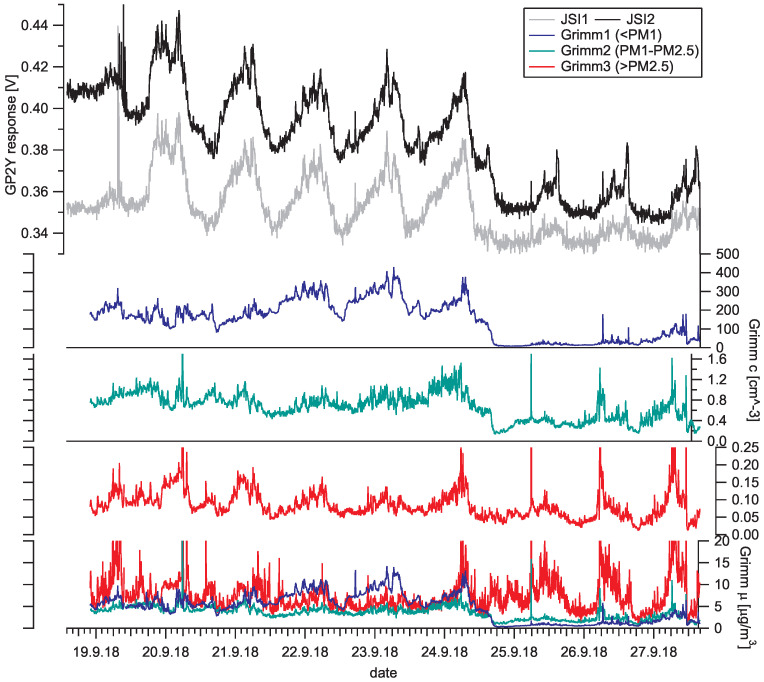
Comparison of Sharp low-cost sensors response and Grimm OPC particle number concentrations during one week of simultaneous measurement on a busy street in Cassino, Italy. Five-minute averages are shown. The two GP2Y sensors (top two curves) are denoted JSI1 and JSI2. Grimm 1, 2 and 3 labels correspond to PM diameter intervals d<1μm, 1μm≤d≤2.5μm and d>2.5μm, respectively. In the middle three graphs, particle number concentrations are shown (in particles per cubic centimeter), and the bottom graph displays the corresponding measured mass concentrations (in micrograms per cubic meter).

Using low-cost optical PM sensors in real environmental measurements is of increasing interest to the aerosol community. Their suitability and limitations are still to be determined. Their sensitivity to environmental parameters (humidity, temperature, light), the stability and degradation over time are still not known thoroughly. In the scope of the AEROMET project [25], we have performed field measurements on 18–27 September 2018 in Cassino, Italy [26]. We sampled ambient aerosol near a busy street with several different types of aerosol monitoring instruments, including two identical Sharp low-cost aerosol sensor devices (referred to as JSI1 and JSI2) and Grimm Portable Laser Aerosol Spectrometer Model Mini-LAS 11-R. The data from these three detectors are shown in Figure 9, where the data from Grimm optical particle spectrometer are joined into three bins: smaller than PM1, PM1–PM2.5 and larger than PM2.5 for clarity. The daily cycles of PM concentrations are clearly observed, as well as a dip in small particle concentration after the rain on 24 September.

**Figure 10:**
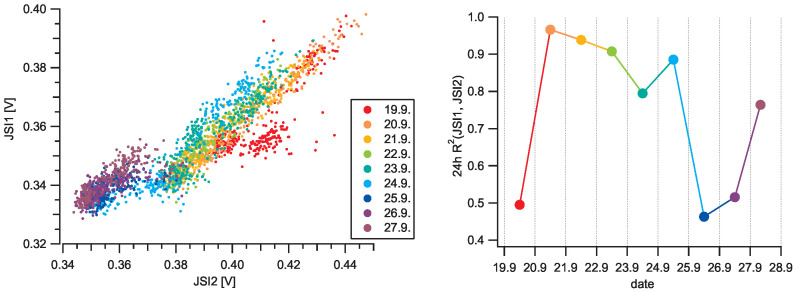
(**Left**) correlation between two identical devices using Sharp GP2Y sensors acquiring data simultaneously on the same spot during the environmental measurements (Figure 9). Each point corresponds to a five-minute average of the sensor’s output. While the correlation is clear ($R^{2}$ = 0.77), both sensors do not have the same calibration and a time drift can clearly be observed. (**Right**) the coefficient of determination $R^{2}$ in 24-h intervals on different days.

To investigate the repeatability of the GP2Y sensor, we correlated the outputs of both low-cost sensors averaged over five minutes (Figure 10). The coefficient of determination calculated for the whole duration of the campaign (eight days) is $R^{2}$ = 0.77. Observing the correlations in 24-h windows, the coefficient varies $R^{2}$ from 0.49 up to 0.97. Two identical GP2Y sensors being placed next to each other follow the general trend well; however, they do differ in their response and must be individually calibrated. Moreover, we were able to observe slow temporal drift in correlation, which can be seen in Figure 10, where light output of the JSI2 device slowly decreased over a week. The exact source of this drift is unknown to us. The manufacturer does warn about LED diode degradation of 50%/5 years [15]; however, this is a very slow process. It also worth noting that no regulated airflow was used on the GP2Y sensors and natural diffusion of particles to the sensitive area was relied upon, so certain deviations between the sensors are expected [9].

**Figure 11:**
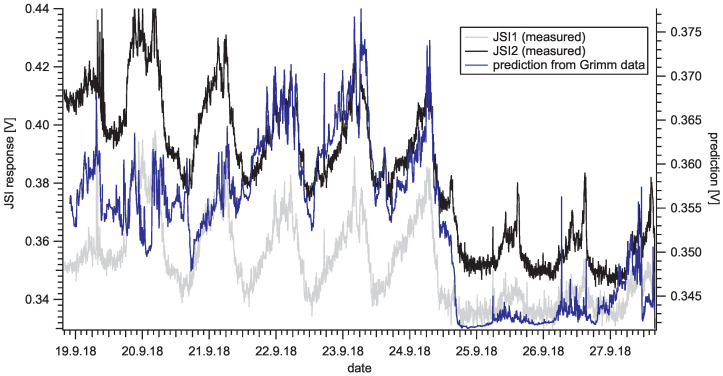
Sensor output in an environmental measurement and comparison with predicted output calculated from particle distribution measurement with OPC Grimm device (JSI1 $R^{2}$ = 0.47, JSI2 $R^{2}$ = 0.58). The data time resolution is five minutes. The JSI1 signal is shown just for information, since it was not calibrated.

We compared the response of the Sharp low-cost sensors with the Grimm particle counter (Figure 9). The Grimm instrument reported aerosol particle number concentrations sorted by diameter ($c_{d}$) from 0.25–32 μm; this information was used to predict Sharp GP2Y sensor response according to Equation (7), taking $U_{0}$ = 0.340 V, *a* = 0.321 mV·cm${}^{3}$ and *n* = 1.1. Since the majority of the PM particles had a diameter *d* < 1 μm, the predicted response is very similar to the numeric (or mass) concentration of small PM denoted Grimm1 in Figure 9. The agreement between the predicted and actual sensor response is shown in Figure 11. One can observe the overall scaling factor between the real response and prediction, which is attributed to differences between measured aerosol material (ambient) and material used for calibration (PSL). There seems to be a time interval where agreement is much better (from 12:00 p.m., 21 September to 12:00 p.m., 24 September), an interval where agreement is good with different scaling (up to 12:00 p.m., 21 September) and worse agreement (from 12:00 p.m., 24 September to the end). Inverse transformation, i.e., to obtain the size distribution information from the Sharp GP2Y output signal value requires prior assumptions about PM distribution and is beyond the scope of this work.

## 5. Conclusions

Low-cost sensors are an intriguing topic in modern aerosol science. Their simplicity and availability make them attractive to professional and general public, where they are widely used in “smart” IOT devices. Their suitability for PM monitoring and their limitations are still being discussed. The proposed approach with digitising output pulses of a sensor is useful in the characterisation stage, where the data can be saved and thoroughly analysed offline later. Then, relevant parameters can be identified and optimal real-time algorithm can be developed based on the hardware limitations. The output of PM sensors, such as Sharp GP2Y, with the output pulse bandwidth in the order of 10 kHz can easily be digitally analysed in real time with simple commercial microcontrollers, and energy efficient battery powered low-cost PM sensing devices can be built around them. To analyse and understand the sensor’s response to different particle types, sizes and concentrations, appropriate infrastructure for generating such controlled environment is needed. To further characterise the simple sensor we used in this work, more different monosized particles measurements would be beneficial to determine the full detectable range of PM diameters and sensor’s response to different types of particles (albedos). After having the sensor fully characterised and being able to predict its output on known input, the inverse calculation (with some assumptions) can be employed to extract more data from given output when the sensor is employed in an ambient atmosphere. A valuable lesson in the low-cost sensor characterisation was an observed discrepancy between the manufacturer documentation and the device’s behaviour. Low-cost sensors with lacking manufacturer’s support should therefore not be taken simply as black-boxes and every step in their usage should be critically evaluated and verified, including the individual adjustment of each particular sensor instance.

## Figures and Tables

**Table 1:** Parameters to describe the data in Figure 6.

	Mean [mV]	Std. Dev. [mV]	CV[%]
Clean box, fixed	314.9	72.8	≈22
Clean box, parabola	332.0	75.7	
Clean box, model	338.9	79.6	
Vacuum, fixed	331.5	63.0	≈16
Vacuum, parabola	349.1	58.1	
Vacuum, model	355.0	65.9	
0.4 μm, fixed	780.5	78.2	≈10
0.4 μm, parabola	794.3	78.5	
0.4 μm, model	798.3	79.2	

**Table 2:** Parameters to describe the data in Figure 8.

*d* [μm]	$b_{d}$ [mV cm${}^{3}$]	$U_{0}$ [mV]	$k_{m,d}$ [mV m${}^{3}$/μg]	$R^{2}$	LOD${}_{500}$ [μg/m${}^{3}$]
0.43	0.127 ± 0.002	343.9 ± 1.4	2.90 ± 0.11	0.995	2.5
1.0	0.328 ± 0.003	346.6 ± 1.5	0.597 ± 0.010	0.985	16
4.3	1.681 ± 0.035	264.5 ± 6.5	0.0385 ± 0.0010	0.951	250
*n*		*a* [mV cm${}^{3}$]		$\tilde{a}$ [mV m${}^{3}$/μg]	
1.12 ± 0.06		0.328 ± 0.017		0.597 ± 0.030	

**Table 3:** Literature review of GP2Y comparison to other instruments.

Reference	Comparison to	Slope	$R^{2}$	Comment
Wang et al. [9]	another GP2Y		0.97	Incense particles
	Sidepack		0.99	0–1000 μg/m${}^{3}$
Manikonda et al. [10]	APS 3321	3.1	0.45	Four calibrated devices based on GP2Y sensor.
	APS 3321	7.7	0.99	
	APS 3321	2.8	0.42	
	APS 3321	1.6	0.85	
Sousan et al. [11]	APS 3321	0.9–1.3	0.95–0.99	compared calibrated GP2Y output
Liu et al. [13]	TEOM	0.99 mV m${}^{3}/\mu$g		95 nm NaCl
		1.00 mV m${}^{3}/\mu$g		86 nm NaCl
		0.53 mV m${}^{3}/\mu$g		70 nm NaCl
Li et al. [12]		5–10 μg/m${}^{3}$/UA		arb. analog units in slope
Li et al. [5]	Sidepack		>0.99	
Hapidin et al. [17]	HPMA115S0	2 mV m${}^{3}/\mu$g	0.956	Incense particles, $d_{\mathrm{mode}}$ = 300 nm,
	TSI 3025A	0.051 mV cm${}^{3}$	0.960	
this work	Welas Digital 2100	2.9 mV m${}^{3}/\mu$g	0.995	PSL, *d* = 430 nm
		0.6 mV m${}^{3}/\mu$g	0.985	PSL, *d* = 1 μm
		0.04 mV m${}^{3}/\mu$g	0.951	PSL, *d* = 4.3 μm
